# Cryo-EM structure of the dimeric *Rhodobacter sphaeroides* RC-LH1 core complex at 2.9 Å: the structural basis for dimerisation

**DOI:** 10.1042/BCJ20210696

**Published:** 2021-11-09

**Authors:** Pu Qian, Tristan I. Croll, Andrew Hitchcock, Philip J. Jackson, Jack H. Salisbury, Pablo Castro-Hartmann, Kasim Sader, David J.K. Swainsbury, C. Neil Hunter

**Affiliations:** 1 Materials and Structural Analysis, Thermo Fisher Scientific, Achtseweg Noord 5, 5651 GG Eindhoven, Netherlands; 2 School of Biosciences, University of Sheffield, Sheffield S10 2TN, U.K.; 3 Cambridge Institute for Medical Research, University of Cambridge, Cambridge CB2 0XY, U.K.; 4 Department of Chemical and Biological Engineering, University of Sheffield, Sheffield S1 3JD, U.K.

**Keywords:** bacteriochlorophylls, carotenoids, light harvesting, photosynthesis, reaction centre, transmembrane proteins

## Abstract

The dimeric reaction centre light-harvesting 1 (RC-LH1) core complex of *Rhodobacter sphaeroides* converts absorbed light energy to a charge separation, and then it reduces a quinone electron and proton acceptor to a quinol. The angle between the two monomers imposes a bent configuration on the dimer complex, which exerts a major influence on the curvature of the membrane vesicles, known as chromatophores, where the light-driven photosynthetic reactions take place. To investigate the dimerisation interface between two RC-LH1 monomers, we determined the cryogenic electron microscopy structure of the dimeric complex at 2.9 Å resolution*.* The structure shows that each monomer consists of a central RC partly enclosed by a 14-subunit LH1 ring held in an open state by PufX and protein-Y polypeptides, thus enabling quinones to enter and leave the complex. Two monomers are brought together through N-terminal interactions between PufX polypeptides on the cytoplasmic side of the complex, augmented by two novel transmembrane polypeptides, designated protein-Z, that bind to the outer faces of the two central LH1 β polypeptides. The precise fit at the dimer interface, enabled by PufX and protein-Z, by C-terminal interactions between opposing LH1 αβ subunits, and by a series of interactions with a bound sulfoquinovosyl diacylglycerol lipid, bring together each monomer creating an S-shaped array of 28 bacteriochlorophylls. The seamless join between the two sets of LH1 bacteriochlorophylls provides a path for excitation energy absorbed by one half of the complex to migrate across the dimer interface to the other half.

## Introduction

Charge separation in the reaction centres (RCs) of phototrophic bacteria relies on light absorbed by a surrounding light-harvesting complex 1 (LH1) antenna, which adopts one of several strategies to allow quinones and their reduced quinol products to enter and leave the complex. The simplest of these is represented by the 16-subunit LH1 ring round the RC (RC-LH1_16_) found in bacteria such as *Thermochromatium tepidum* and *Rhodospirillum rubrum*, which completely encloses the RC. Each of the LH1 subunits binds one carotenoid, leaving small pores between LH1 subunits for quinone diffusion [1–3]. Next, bacteria such as *Rhodobacter (Rba.) veldkampii* and *Rhodopseudomonas palustris* retain these pores and also have an extra transmembrane polypeptide (PufX or protein-W respectively) that interrupts the LH1 ring, preventing it from completely encircling the RC [4,5]; the resulting gap provides a straightforward entry and exit point for quinone diffusion. Finally, in the monomeric RC-LH1_14_–PufX complex of *Rba. sphaeroides*, each LH1 subunit binds two carotenoids, enhancing light harvesting and photoprotection but hindering quinone diffusion through the small pores within the LH1 ring [6]. The gap in the LH1 ring created by PufX then becomes crucial for photosynthesis [7–9] because it is the only route for efficiently channelling quinones across the LH1 ring. Possibly in response to this single opening in LH1, PufX is augmented by the transmembrane protein-Y, which inserts between the LH1 and RC complexes near the RC Q_B_ site. Protein-Y binds to the internal surface of LH1 and its RC-facing side leaves an internal channel through which quinols and quinones can pass [10].

The 2.5 Å resolution structure of the monomeric RC-LH1 core complex reveals the molecular interactions that enable this complex to perform light harvesting, photochemistry and quinol export [10]. The structure also shows how PufX carries out its chief function, which is to prevent LH1 subunits completely surrounding the RC, allowing quinols and quinones to diffuse rapidly between the RC and the cytochrome *bc*_1_ complex [9,11]. However, this polypeptide performs other important roles; PufX is a plausible candidate for initiating the progressive encirclement of the RC by LH1 subunits [12], each of which consists of a pair of single transmembrane spanning α and β polypeptides [10]. In this oligomerisation process, the RC is a template that guides the assembly of a curved LH1 array [13]. PufX is also required for dimerisation of the RC-LH1 complex [14–18], and while dimers are not essential for photosynthesis, they adopt a bent configuration [19,20] that imposes curvature on the photosynthetic membranes of *Rba. sphaeroides* [21]. Mutants that produce RC-LH1 dimers in the absence of the peripheral LH2 antenna have tubular membranes [19,21–23] whereas the wild-type has spherical intracytoplasmic membrane vesicles, termed chromatophores, due to the presence of LH2 complexes [24–27]. The local membrane curvature imposed by bent RC-LH1 dimers appears to create a favourable environment for stable assembly of LH2 complexes [28], which also confer curvature on membranes [24]. Given the importance of PufX for dimerisation of the RC-LH1 complex, we previously used X-ray crystallography to obtain an 8 Å resolution structure of the dimer [20]. The basic architecture of the complex was apparent at this resolution, including some interactions at the monomer–monomer interface, but many important aspects of the complex were not resolved, including additional polypeptide subunits and the carotenoids [20]. Here, we report the structure of the dimeric RC-LH1 complex, determined by cryogenic electron microscopy (cryo-EM), at a resolution of 2.9 Å. This structure shows the relative orientations of the two PufX polypeptides as they incline towards each other at the monomer–monomer interface, and it reveals four copies (two per monomer) of a hitherto unknown transmembrane polypeptide, protein-Z, which was not found in the RC-LH1 monomer. Finally, our structure shows how the C-terminal PufX residues Arg49 and Arg53, together with a sulfoquinovosyl diacylglycerol (SQDG) lipid, contribute to binding two monomers together to subtend a 152° angle, which curves the membrane bilayer in which dimers are embedded.

## Materials and methods

### Protein purification

Dimeric core complex was extracted from a LH2-deficient strain, DBCΩG, of *Rba. sphaeroides* [20]. The cells were cultured in M22+ medium under semi-aerobic conditions in darkness (1.4 L M22+ medium in 2 L conical flask shaken at 150 rpm at 30°C). Cells were harvested by centrifugation at 4 000×*g* for 30 min when the culture reached an optical density of 1.6 at 680 nm and were stored at −80°C before use. Purification of the dimeric RC-LH1 complexes was performed as described previously [20]. In brief, washed cells were suspended in working buffer (20 mM HEPES, pH 7.8) and broken three times in a French Press (AminCo, U.S.A.) under a pressure of 18 000 psi. The broken cells were loaded on a two-step sucrose gradient (15/40% (w/v) sucrose in working buffer). After 4 h of centrifugation at 100 000×*g* photosynthetic membranes, located above the 40% sucrose layer, were collected. This fraction was diluted three times using working buffer and pelleted by centrifugation for one hour at 235 000×*g*. The membranes were re-suspended and homogenised in a small amount of working buffer and the final absorbance of the membrane at 874 nm (A_874_) was adjusted to ∼100. For protein solubilisation, the concentration of the membrane was adjusted to an A_874_ of 60 before the addition of *n*-dodecyl β-d-maltoside (β-DDM) to 3% (w/v). The solution was stirred at 4°C for 30 min and insoluble material was removed by centrifugation for one hour at 211 000×*g*. The pigmented supernatant was applied to continuous sucrose gradients (20–40% (w/w) sucrose in running buffer — working buffer containing 0.03% (w/v) β-DDM) and centrifuged at 197 000×*g* for 20 h. A major band at ∼30% sucrose containing dimeric core complexes was collected and purified on a DEAE ion exchange column. After loading the sample, the column was washed using running buffer containing 100 mM NaCl. A gradient from 100 to 300 mM NaCl was used to elute the complexes from the column. The dimer band eluted at ∼250 mM NaCl and fractions with an A_874_/A_280_ ratio of >1.6 were pooled and concentrated, then applied to a size exclusion column (Superdex 200 16/60 GL, GE Healthcare) pre-equilibrated in working buffer. Fractions eluting from the size exclusion column with a ratio of A_874_/A_280_ >1.9 were used for cryo-EM grid preparation.

### Data collection

The protein solution was concentrated to an A_874_ of 74. The cryo-EM grid was prepared using an FEI Vitrobot MK4 with the following parameters: sample chamber temperature, 4°C; sample chamber humidity, 99%; blotting time, 2.5 s; blotting force, 3. Protein solution (3.0 µl) was applied to the EM grid (QuantiFoil R 1.2/1.3, 300 mesh Cu, glow discharged for 60 s under 25 mA using an easiGlow glow discharger (Ted Pella. Inc)). Following incubation and blotting, the grid was plunged into liquid ethane cooled by liquid nitrogen and stored under liquid nitrogen until required.

Cryo-EM data were collected on a Titan Krios2 G3i cryo electron microscope (Thermofisher Scientific) equipped with a Falcon 4 direct electron detector at the Cambridge Pharmaceutical Cryo-EM Consortium [29]. The microscope was operated at 300 kV accelerating voltage and a nominal magnification of 120 k, corresponding to a pixel size of 0.65 Å at the specimen level. The detector was operated in counting mode. A total dose of 45.36 electrons per Å^2^ was fractionated to 42 frames within a 12.2 s exposure time, resulting in an electron dose of 1.08 e^−^/Å^2^/frame. In total, 5085 movies were collected with defocus values ranging from −0.8 to −2.2 µm. A typical cryo-EM image, averaged from motion corrected movie frames, is shown in Supplementary Figure S1A.

### Data processing

Beam induced motion correction of micrographs was performed using RELION's [30] implementation of Motioncor2 [31] with 5 × 5 patches. CTF correction was performed using CTFFIND4 [32]. Particle coordinates on the motion corrected images were initially obtained using cisTEM [33], and were subsequently manually adjusted. Dimeric core complexes were ∼20 × 10 nm. A box size of 470 pixels, corresponding to a 30.5 nm square at the specimen level, was used for particle extraction. In total, 223 786 particles were picked. The particles were subjected to reference free 2D classification. 161 454 (72%) particles in good 2D classes (see Supplementary Figure S1B) were selected for 3D classification. An initial model for 3D classification was produced *de novo* in RELION. The best 3D model out of four, containing 58 945 (26%) particles, was selected for high resolution 3D reconstruction. At this stage, C2 symmetry was imposed, resulting in a 3.35 Å resolution 3D map. After CTF refinement, including anisotropic magnification, beam-tilt, trefoil, 4th order aberration, per-particle defocus and per-image astigmatism estimation, the resolution of the 3D map of the dimeric complex was enhanced to 3.15 Å. Bayesian polishing was executed with the default parameters provided by RELION on re-extracted particles in a box of 512 × 512 pixels to produce a final 3D map at 2.87 Å resolution.

### Refinement and modelling

Two monomeric RC-LH1 complexes (PDB 7PIL) were docked into the 2.87 Å resolution dimer map as rigid bodies using ChimeraX [34]. The spheroidene carotenoids were replaced by methoxy-neurosporene in keeping with the carotenoid content of the mutant used here. Four extra transmembrane helices (two per monomer) were identified in the map; these were initially traced as poly-alanine in COOT [35]. Comparison of density around each chain revealed they were almost certainly identical in sequence, and we designated them as protein-Z. Preliminary assignment of putative sidechains was performed in ISOLDE [36], considering both fit to density and local environment for the best-resolved chain. A BLASTP search of the resulting sequence against *Rba. sphaeroides* using the NCBI server yielded a series of related single-pass transmembrane sequences with near-identical sequence in their N-terminal transmembrane helices. The final assigned sequence was identified by mass spectrometry as described below, and modelled into the density with ISOLDE. One complete monomer was then thoroughly inspected and rebuilt in ISOLDE, revealing a very clearly resolved SQDG lipid at the dimer interface, with its sulfonic acid complexed to Arg49 and Arg53 of PufX. This is packed against another unusual lipid; while the density is incompatible with diacylglycerol phospho- or glycolipids, ultimately we were unable to identify it from the density and opted to leave it unmodelled. Similarly, we found an elongated density feature inserted between PufX, RC-L and two LHα chains, which could be an extra methoxy-neurosporene carotenoid but again it was left unmodelled. Once remodelling of the monomer was complete, strict symmetry was reimposed by replacing the second monomer copy with the updated and extended coordinates. Finally, the complete model was refined in phenix.real_space_refine [37].

### Identification of protein-Z by mass spectrometry

Pure RC-LH1 core complex (50 µg) was solubilised and digested with pepsin, and the resulting peptides were analysed by nano-flow liquid chromatography coupled to a Q Exactive HF quadrupole-Orbitrap (Thermo Scientific) mass spectrometer as described in our previous work [10,38]. Protein identification was performed by database searching using Byonic (v2.9.38, Protein Metrics) operating with the default parameters except that methionine sulfoxide (+15.19949 Da) was specified as a common variable modification (with a maximum of two per peptide). Cleavage sites were specified as N- and C-terminal to F, Y, W and L (semi-specific). The database was the *Rba. sphaeroides* reference proteome (www.uniprot.org/proteomes/ UP000002703) downloaded on 28 April 2021 and edited by adding the putative protein-Z sequence derived by the re-annotation of the Rsp_2385 locus (see Supplementary Figure S2).

## Results and discussion

### Overall structure of the RC-LH1 dimer complex

**Figure 1.**
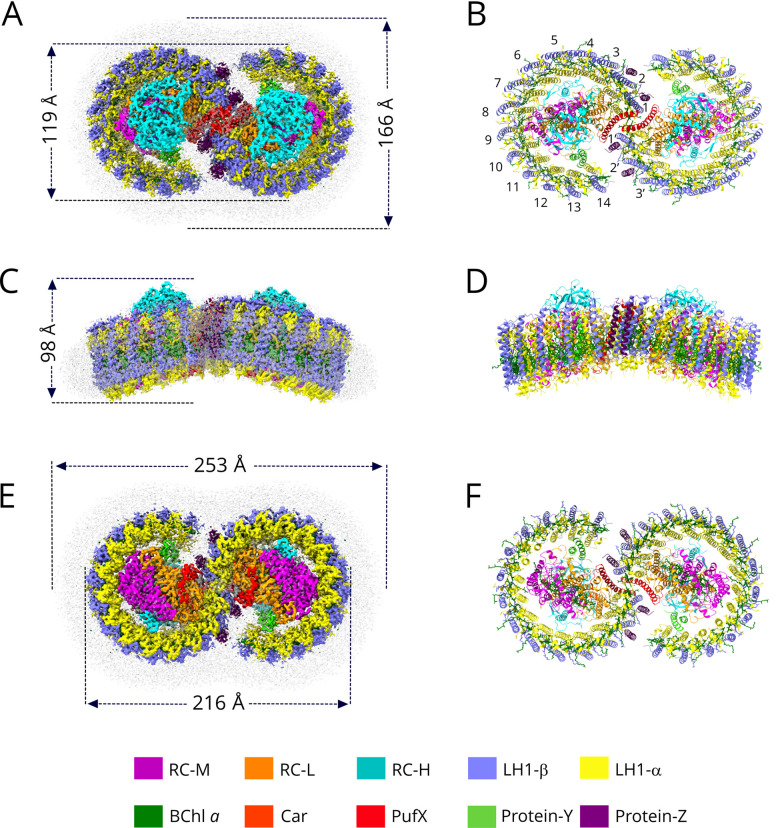
Cryo-EM structure of the dimeric RC-LH1 complex from *Rba. sphaeroides*. (**A**,**C**,**E**) Views of the density map, coloured as in the key at the bottom of the figure. Detergent and other disordered molecules are in grey. (**B**,**E**,**F**) Ribbon models of the complex, made using ChimeraX [34]. (**A**) View of the cytoplasmic face of the density map of the complex, showing the diameters of the short axes of the detergent belt and the complex. (**B**) View as in (**A**), as a ribbon model; the LH1 subunits are numbered. (**C**) View of the density map in the plane of the membrane showing the height of the complex. (**D**) View as in (**C**), as a ribbon model. (**E**) Perpendicular view of the density map from the periplasmic side with measurements of the long-axis of the complex and detergent micelle. (**F**) Ribbon model corresponding to (**E**).

The dimeric RC-LH1 complex was purified from a LH2-deficient strain of *Rba. sphaeroides* harbouring a *crtD* mutation, in which methoxy-neurosporene is the major carotenoid [39]. This differs from spheroidene made by the wild-type strain in one respect, having a single C3–C4 bond rather than a double bond. Assembly of dimeric core complexes is strongly favoured in both *crtD* and *crtA* (spheroidene only) strains [39]. A typical cryo-EM image of the RC-LH1 dimer complex is shown in Supplementary Figure S1, together with selected 2D classes and the Fourier shell correlations. Supplementary Table S1 displays the information on data acquisition, model refinement and validation statistics. Supplementary Figure S3 shows the fits of structural models of polypeptides, pigments and lipids of the RC-LH1 dimer complex within their respective cryo-EM densities. The 2.9 Å structure of the dimeric RC-LH1 complex, represented as a series of colour-coded density maps, is shown in Figure 1A,C,E, and structural models of the complex are in Figure 1B,D,F. Enlarged views of Figure 1A,B are shown in Supplementary Figure S4. Each half of the complex consists of an incomplete ring of 14 LH1 subunits, which are numbered in Figure 1B to correspond with the numbers for the monomer complex [10]. The LH1 subunits arc round each RC, leaving a gap between LH1 αβ subunit 14 and PufX. The two transmembrane helices of protein-Y sit inside the LH1 ring, against the surface formed by the α polypeptides belonging to LH1 subunits 13 and 14. The main feature of the dimer is the concave periplasmic surface brought about by the association of two monomers, subtending an angle of 152° (Figure 1C,D). This is comparable with the 146° angle measured using EM of negatively stained dimer complexes [19] and 158° derived from the 8.0 Å structure determined by X-ray crystallography [20]. In the latter case, the 3D crystals obtained consisted of ordered stacks of 2D crystals, and the packing of dimers in the lattice flattened the complexes slightly, hence the 158° angle. This small variation could indicate some limited flexibility at the monomer−monomer interface, a conclusion further supported by atomic force microscopy (AFM) of membrane-embedded dimer complexes adsorbed onto a mica surface [40].

### A new component in the dimeric core complex: protein-Z

Superposition of the monomeric RC-LH1 structure [10] onto one half of the dimer (not shown) indicates an almost exact correspondence (RMSD of 0.177 Å) between the structures. Thus, all of the structural and functional aspects of the monomer, in particular regarding the roles of PufX and protein-Y in creating a channel for fast quinone diffusion, are retained in the dimer complex. Here, we focus on those aspects of the structure directly relevant to dimerisation, and we found that the dimer comprises more than just two monomers. Figure 1 shows the surprising presence of two single transmembrane polypeptides, sitting on the outer face of the LH1 β1 and β2 polypeptides. We assigned a tentative *de novo* sequence on the basis of the best-resolved regions of the unknown helices by adding side-chains in ISOLDE, considering both fit to density and physical environment; we previously used the same approach for identification of protein-Y in our monomer structure [10]. The tentative sequence was used as the basis for a BLASTP search of *Rba. sphaeroides*, identifying the N-terminal transmembrane helix of a 102 amino acid protein with a molecular mass of 10 564 Da as the best candidate; we provisionally name this component as protein-Z.

We were initially unable to identify the open reading frame coding for this protein in the *Rba. sphaeroides* 2.4.1 strain used in this work, but upon inspection of the re-annotated genome (available here: https://www.genome.jp/dbget-bin/www_bget?refseq+NC_007493) we found that the putative gene encoding protein-Z (designated as *puzA*) is located upstream of Rsp_6224 and downstream of Rsp_2384, with the three genes all transcribed in the same direction (Supplementary Figure S2A). Note that in the original *Rba. sphaeroides* 2.4.1 genome available in the KEGG (Kyoto Encyclopedia of Genes and Genomes) database (https://www.genome.jp/entry/T00284), the gene between Rsp_6224 and Rsp_2384 (locus tag Rsp_2385) is present on the complementary strand and encodes a 120 amino acid hypothetical protein with no homology to protein-Z (Supplementary Figure S2B). The corresponding *puzA* gene also seems to be misannotated in the three other *Rba. sphaeroides* genomes available in the KEGG database, namely those of *Rba. sphaeroides* strain ATCC 17025 (https://www.genome.jp/entry/T00512), *Rba. sphaeroides strain* ATCC 17029 (https://www.genome.jp/entry/T00484) and *Rba. sphaeroides* strain KD131 (https://www.genome.jp/entry/T00844). The predicted proteins from the ATCC 17029 and KD131 strains are very similar to those from *Rba. sphaeroides* 2.4.1 (100/102 and 101/102 residues identical, respectively), whereas the 101 amino acid protein from *Rba. sphaeroides* ATCC 17025 is only homologous in the N-terminal transmembrane region, with 23 of the first 29 residues conserved (Supplementary Figure S2C).

Mass spectrometry was used to analyse a pepsin digest of purified RC-LH1 core complexes. In addition to the RC-H, RC-M, RC-L, PufX, protein-Y, LH1α and LH1β polypeptides identified in our previous work [10], the sequences of which are shown in Supplementary Figure S5, we were also able to detect protein-Z by virtue of the C-terminal pepsin 23-mer peptide ADT-KEV (Supplementary Figure S2C). We note that the density map allows only 31 residues to be resolved in our structure (corresponding to the N-terminal 31 residues of protein-Z), thus the position of the rest of the protein, including the C-terminus that we detected by mass spectrometry, requires further study.

**Figure 2.**
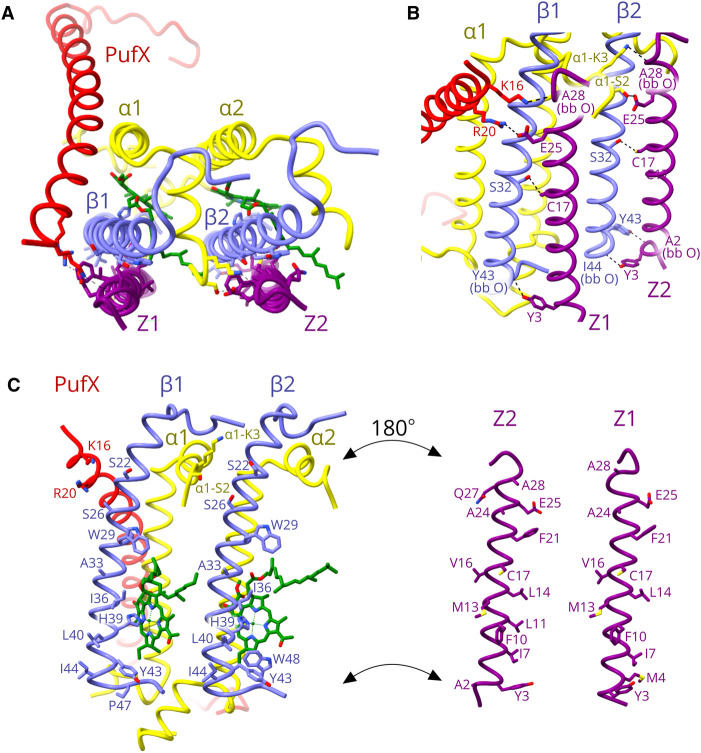
The positions and transmembrane interactions of the two protein-Z polypeptides. (**A**) View of part of the LH1 ring near the monomer–monomer interface, perpendicular to the membrane from the cytoplasmic side. The transmembrane regions of protein-Z1 and protein-Z2 are closely appressed against the LH1 β1 and β2 polypeptides. (**B**) View in the plane of the membrane, showing hydrogen bonds (dashed lines) between Z1/Z2 and the respective β1 and β2 polypeptides. Other bonds with LH1 α1 and PufX near the cytoplasmic face of the membrane are labelled. Supplementary Table S2 lists the hydrogen bonds relating to Figure 2B. (**C**) ‘Open book’ format to show the opposing, interacting hydrophobic faces of Z1 and Z2 and the respective β1 and β2 polypeptides. The labelled residues are predicted to be in van der Waals contact.

The absence from the monomer complex indicates that the protein-Z polypeptides are connected in some way to dimer formation, and their location near the dimer interface (Figs. 1, 2A) is consistent with this suggestion. Figure 2A shows that the transmembrane regions of Z1 and Z2 are held tightly against the outer face of the LH1 β1 and β2 polypeptides. Figure 2B,C examine the nature and extent of the bonds that hold these polypeptides in place; Z1 and Z2 have extensive hydrogen bonding and hydrophobic interactions with LH1 β1 and β2 in particular, but also with LH1 α1 and PufX near the cytoplasmic face of the membrane. Supplementary Table S2 lists the hydrogen bonds relating to Figure 2B.

### Protein–protein and pigment–protein interactions that hold the two monomers together

**Figure 3.**
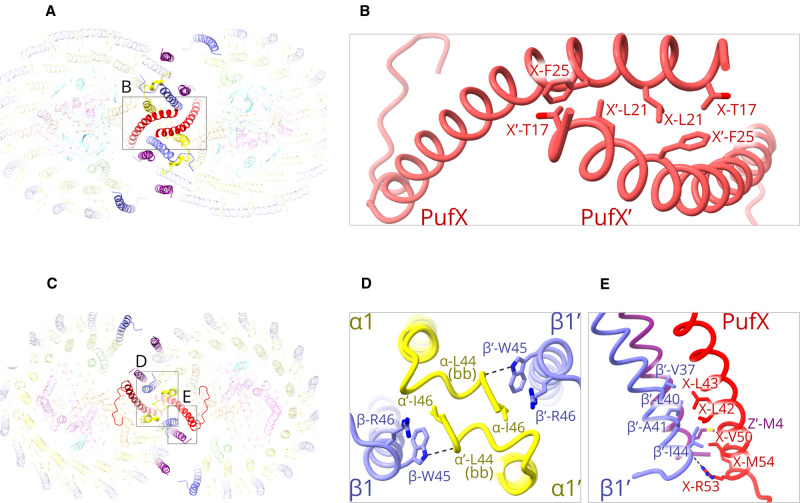
Protein–protein interactions at the dimer interface. (**A**) View of the cytoplasmic side of the dimer. For clarity, all components are faded except for those at the interface. (**B**) Detailed view of the region indicated by the box in (**A**) showing PufX and PufX’ interacting near their N-termini on the cytoplasmic side of the membrane, with important sidechains labelled. (**C**) View of the periplasmic side of the dimer, with other features as in (**A**). (**D**) Detailed view of the region indicated by the left-hand box in (**C**), and rotated by 90°, showing C-terminal interactions between opposing LH1 α, α′ and β, β′ subunits at positions 1 and 1′ (see Figure 1B for numbering). Dashed lines indicate hydrogen bonds, which are 3.3 Å (see also Supplementary Table S2). (**E**) The components shown in the right-hand box in (**C**), but viewed from a different angle, showing C-terminal interactions between PufX on one side of the dimer complex, and protein-Z′ and the LH1 β′ subunit at position 1′ on the other side. The hydrogen bond between X-Arg53 and β1′-Ile44 is 2.8 Å (see also Supplementary Table S2).

PufX is important for quinone diffusion to and from the RC Q_B_ site, and for promoting dimerisation. The structure of the RC-LH1 monomer complex [10] clearly showed how a network of hydrogen bonds anchors the C-terminal region of PufX to the RC-L subunit on the periplasmic side of the complex, and the same is found for the dimer complex (not shown). Then, moving towards the N-terminus, PufX crosses the first LH1 αβ subunit, making several hydrophobic contacts with the LH1 transmembrane regions before emerging on the cytoplasmic side of the membrane. Here, we focus on those interactions that determine dimerisation. As Figure 3B shows, the two opposing PufX polypeptides, labelled X and X’, are tilted towards each other so although their transmembrane regions are distant from one another their respective N-terminal regions are brought together, starting with residues 17–25 (Figure 3A,B). The density map did not resolve the 14 N-terminal residues of PufX; this information would have shed light on the role of this region in dimerisation because it has been shown that the first 12 residues of PufX are essential for dimerisation [16].

To provide some indications of the disposition of the N-terminal residues absent from PufX in the dimer structure, we used the solution structures of PufX determined previously by NMR, which do have intact N-termini [41]. Supplementary Figure S6 shows some of the many solution structures of PufX, aligned to the full length of the transmembrane helix, with the PufX structure from the dimer included for comparison. The large variation in conformation of the C-terminal domain reflects the lack of structural constraints in solution, but constraints are imposed when PufX is incorporated within the RC-LH1 complex. The same applies to the widely varying N-terminal domains, of which one, coloured in blue in Supplementary Figure S6, aligns to a limited degree with the PufX assembled within the dimer complex. We conclude that the unresolved N-terminal residues are likely to have some conformational flexibility, possibly leading to further interactions with the opposing complex, for example with the unresolved C-terminal region of the RC-H subunit.

The periplasmic side of the complex is shown in Figure 3C, and two areas have been selected for a more detailed view. Figure 3D shows how opposing LH1 α, α′ and β, β′ subunits at positions 1 and 1′ form a series of bonds, including a hydrogen bond between the backbone oxygen of α-Leu44 and the sidechain of β-Trp45. Thus, β1-α1′ and β1′-α1 bonds bring the LH1 subunits at positions 1 and 1′ closely together, and one consequence is the seamless union of the two rings of LH1 BChls at the interface (see also Figure 6). Further bonds stabilising the dimer on the periplasmic side are shown in Figure 3E. Supplementary Table S2 lists the hydrogen bonds relating to Figure 3D,E. There is one hydrogen bond, between PufX–Arg53 and the backbone oxygen on LH1 β-Ile44, and a series of hydrophobic contacts between the transmembrane regions of PufX and the opposing LH1 β1′, and also with the central protein-Z′ (Z1′). Thus, the Z1 and Z1′ polypeptides appear to contribute to the multiple interactions that stabilise the dimer. The role played by the more peripheral Z2 and Z2′ polypeptides adjacent to LH1 subunits 2 and 2′ is less certain, given that we could only find a single interaction, between Met1 on protein-Z2′ and Phe49 of the opposing β from the second LH1 subunit (not shown). However, given the absence of the majority of the predicted protein-Z sequence (Supplementary Figure S2C) from our density map, other stabilizing interactions may occur.

### Lipid–protein interactions on the periplasmic side of the complex

**Figure 4.**
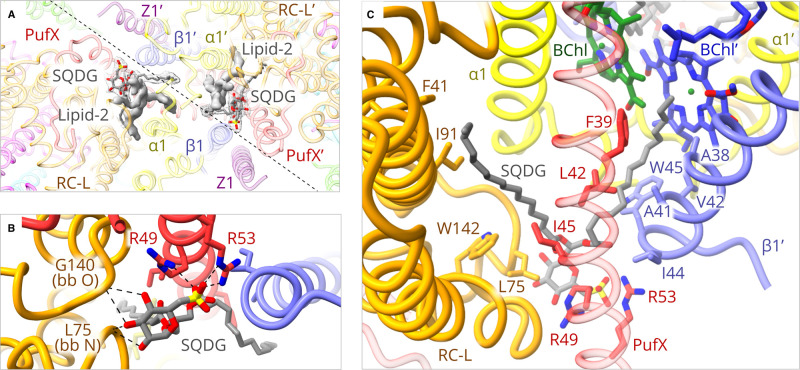
Lipid–protein interactions at the dimer interface. (**A**) View of the interface from the periplasmic side; the diagonal dashed line indicates the approximate position of the interface. For clarity, BChl and carotenoid pigments have been omitted. SQDG lipids are shown within a mesh representing the density; the solid grey densities for another, unassigned, lipid (Lipid-2) are also shown. (**B**) Details of the hydrogen bonds (dashed lines) between the SQDG headgroup and backbone (bb) oxygen or nitrogen, and also with Arg49 and Arg53 near the C-terminus of PufX. Supplementary Table S2 lists the hydrogen bonds relating to Figure 4B. (**C**) View of the dimer interface, showing the disposition of the SQDG lipid tails as they reach up towards the cytoplasmic side of the complex. Sidechains of RC-L, PufX and the opposing LH1β′ subunit that interact with the lipid tails are labelled. For convenience, the numbering of RC-L residues follows the other entries for these RCs in the PDB in omitting the first Met and starting at the second residue (see also Supplementary Figure S5).

In addition to densities for expected pigments and proteins, we found two additional densities that were assigned to lipids. One of these, labelled as Lipid-2 in Figure 4A, could not be assigned with confidence; the density is very strong for the tails, and the shape of the headgroup suggests that it is neither a phospholipid nor a glyceroglycolipid. It is possible that lipid-2 is an ornithine lipid, which has been identified in *Rba. sphaeroides* [42,43]. The excellent fit of the other density indicates the lipid is SQDG [44], also found in dimeric RC-LH1 complexes from *Rba. sphaeroides* solubilised by styrene–maleic acid [45]. Figure 4A–C shows several views of this SQDG that, along with Lipid-2, sits at the interface between the two halves of the dimer, represented in Figure 4A as a diagonal dashed line. Figure 4B shows that the SQDG headgroup forms a series of hydrogen bonds with the backbone of the RC-L subunit, and forms a tightly coordinated salt bridge complex with Arg49 and Arg53 near the C-terminus of PufX. The tails of SQDG snake up through the interface making a number of hydrophobic contacts with sidechains of RC-L and PufX on its own half of the dimer and, crucially, also with the transmembrane region of the opposing LH1β′ subunit (Figure 4C). One of the two SQDG tails also runs across the face of the BChl′ macrocycle on the other side of the complex (Figure 4C). This arrangement is mirrored on the other side of the dimer, with the result that the two SQDG lipids crosslink the dimer halves, held strongly in place by hydrogen bonds on the periplasmic side of the complex, then interacting with the opposing LH1 complex. The many hydrogen bonds to the SQDG head group provided by PufX Arg49 and Arg53 (Figure 4B and Supplementary Table S2) are likely to be essential to bind this lipid at the dimer interface, therefore the present structure helps explain why mutation of either of these residues to Leu abolishes dimer formation [46].

### The structural basis for the bent conformation of the complex

**Figure 5.**
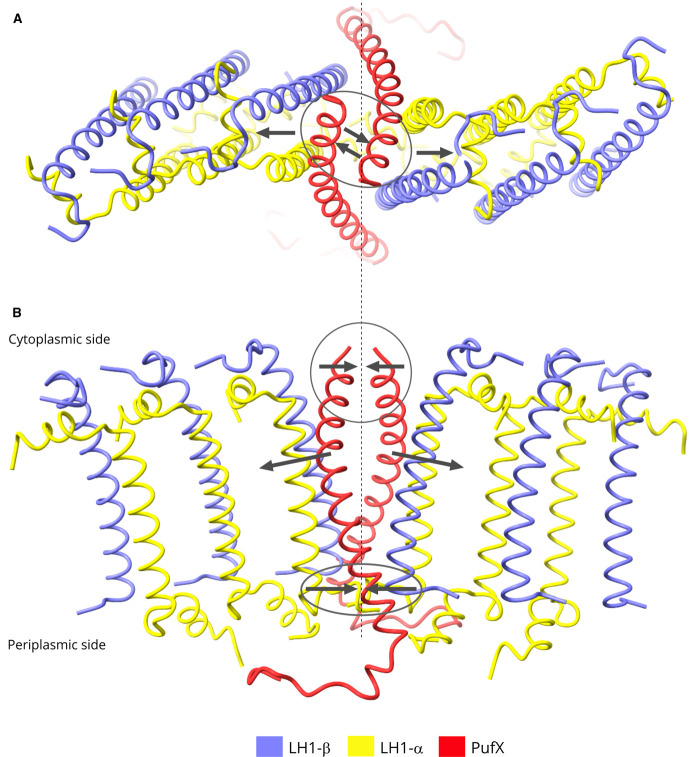
The role of PufX in imposing a bent conformation on the RC-LH1 dimer complex. Pigments and lipids, as well as most of the LH1 subunits and the RC, have been omitted for clarity. (**A**) Top view, from the cytoplasmic side of the membrane. The arrows within the ellipse illustrate the attractive interactions between N-terminal regions of opposing PufX polypeptides, which help to bind the two halves of the dimer together. The diverging arrows represent the effects of each PufX as it lies diagonally across its adjacent LH1αβ subunit, pushing them apart. (**B**) As in (**A**) but viewed in the plane of the membrane, with the N-terminal PufX interactions within the circle, and multiple attractive interactions near the periplasmic face, within the ellipse, that hold the bottom halves of the complex tightly together. As in (**A**) the diverging arrows represent the central LH1αβ subunits being pushed apart by the PufX transmembrane regions.

Figures 2–4 show that many types of interaction hold the dimer together, variously involving protein-Z, PufX, LH1–LH1 contacts, and lipid–protein interactions. There seems to be no obvious reason why the dimer forms a bent structure, yet it is clear from Figure 1 that the association between two monomers locks them together at an angle of ∼152°. Thus, dimer-only membranes are tubular [19,21–23]. Figures 3 and 4 show that the monomer halves are bound tightly together on the periplasmic side of the membrane, and a view in the plane of the membrane (Figure 5) shows that the LH1 subunits open out progressively towards the cytoplasmic side, creating the 152° angle. The transmembrane region of each PufX lies diagonally across its neighbouring LH1αβ subunit, so PufX and X′ act as a brace that enforces separation of the monomers.

**Figure 6.**
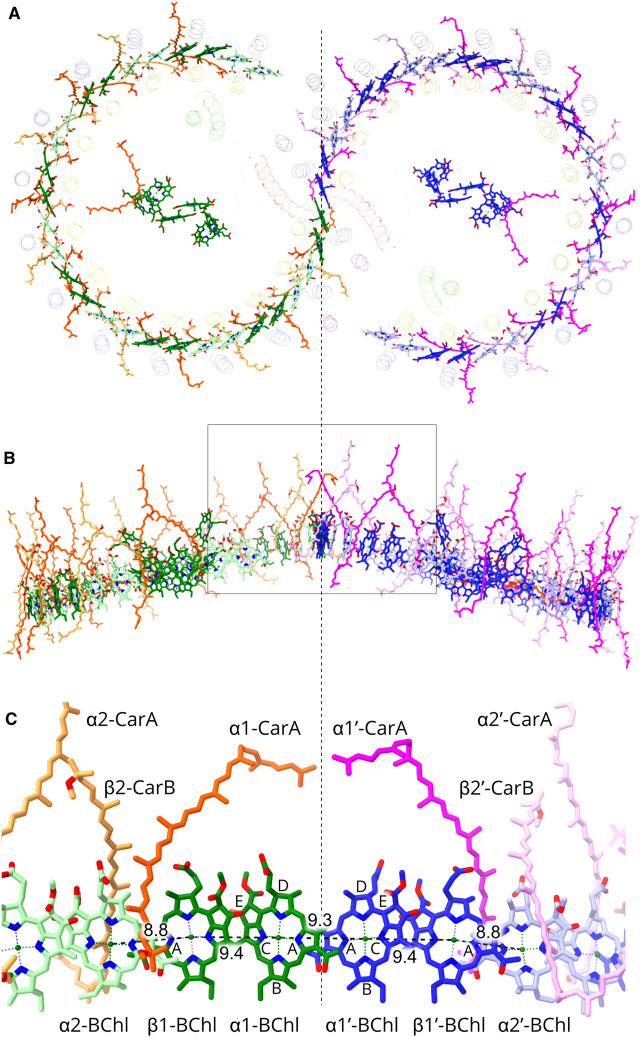
The bacteriochlorophyll and carotenoid pigments in the RC-LH1 dimer. Proteins are faded for clarity. The dashed line divides the complex into the two halves. The central pigments belong to the RCs. (**A**) View from the periplasmic side of the membrane showing the two arcs of 28 BChls. Within one monomer half the BChls are coloured in two shades of green to distinguish between pairs belonging to individual LH1 αβ subunits, and in the other half two shades of blue are used. Similarly, two shades of orange or magenta indicate pairs of carotenoids belonging to individual LH1 αβ subunits on each half of the dimer. (**B**) View in the plane of the membrane. The box indicates the interface zone magnified in panel (**C**). (**C**) BChl and carotenoid pigments at the interface, coloured as in (**A**), and with the α and β polypeptides numbered as in Figure 1B. Rings **A**–**E** of the BChl macrocycles are labelled, and Mg–Mg distances are shown in Ångstroms for intra- and inter-subunit BChls.

One consequence of assembling dimers from two monomers is that the shape changes from a smaller, near-circular complex to a larger, elongated form, and from planar to bent. A Monte Carlo study, augmented by linear dichroism and AFM, compared the tendency of membrane protein structures with different shapes and sizes, in this case RC-LH1 monomers, dimers and LH2 complexes, to aggregate and form domains [26]. It was found that size, shape and curvature differences between these membrane protein complexes create a driving force for assembling curved membranes that segregate into RC-LH1 dimer-rich and LH2-rich domains rather than intermixing [26]. This Monte Carlo simulation replicated the organisation of complexes imaged in native membranes by AFM [25,47]. Thus, dimerisation of the RC-LH1 complex is likely to be an early step in the assembly of photosynthetic membranes of *Rba. sphaeroides*; an AFM study of membranes in the first stages of development identified RC-LH1 dimers [40], and it appears that the dimerisation process is a prerequisite for the subsequent incorporation of LH2 complexes [28]. Other consequences of dimer formation, where they pack along their long axes [47], include excitation transfer, but in addition it was proposed that quinols could transfer along rows of dimers en route to their site of oxidation at cytochrome *bc*_1_ complexes [20]. This is consistent with the confined, quinone-rich regions surrounding RC-LH1 dimers suggested earlier [48].

### Connectivity between the two rings of bacteriochlorophylls

Figure 6A,B shows two views of the two rings of 28 LH1 BChls and the associated carotenoids, which are brought into contact by dimerisation. Ligands to the BChls are the same as those characterised in detail in the structure of the monomer complex [10], with pairs of BChls bound to the LH1 α and β subunits by ligation of their central Mg atoms to α-His32 and β-His39, respectively. Hydrogen bonds are also the same, with the C3 acetyl carbonyl of LH1 α and β BChls hydrogen-bonded to α-Trp43 and β-Trp48, respectively. This combination of bonds holds the Q_Y_ absorption transitions of the BChls, which run from ring A to ring C, approximately parallel to the plane of the membrane (Figure 6C). There are three levels of organisation of the LH1 BChls: within a LH1 αβ subunit, between subunits and, at one position only (α1–α1′; Figure 6C and also see Figure 1B), between rings. Within an LH1 subunit rings C/E of the BChl macrocycles (both the same colour in Figure 4C) overlap to a limited extent, and the A rings of the BChls overlap between all pairs of LH1 subunits in the curved LH1 array (Figure 6C). For the BChls bound to LH1 α1 and α1′ the overlap of A rings is maintained across the join between the two LH1 rings (Figure 6C). The intra-subunit and inter-subunit Mg–Mg distances are 9.4 and 8.8 Å, respectively. At the interface, the BChls of the two LH1 rings are coloured green or blue, and Figure 6C shows the seamless linkage between pigments at this interface, with almost no interruption in the spacing; only 9.3 Å separates the Mg atoms of BChls of the two half complexes at their closest approach (α1 BChl-α1′ BChl; Figure 4C). Figure 6A–C show the arrangement of methoxy-neurosporene carotenoids, of which there are 50; although there are 28 LH1 αβ subunits, those numbered 14 and 14′ have no carotenoid pairs. At the interface, each of the CarB carotenoids (see Figure 6C for carotenoid labelling) is missing because of the proximity to PufX. The CarA carotenoids are prevented from adopting their normal, more linear conformation because they have to lie under the arch formed by the two PufX polypeptides, which incline towards each other (Supplementary Figure S7). Thus, these two central carotenoids are bowed towards each other (Figure 6C). The phytol tails of the two central BChls, α1-BChl and α1′-BChl, are similarly constrained and bowed by PufX and PufX′ (Supplementary Figure S7A).

The detailed structure of the dimer interface shows that the BChls of the two LH1 rings are brought together so precisely that they form a continuous, excitonically coupled chain of pigments, allowing ultrafast migration of energy between the two halves of the complex. Excitation sharing between halves of the dimer was suggested earlier on the basis of kinetics measurements [48], the medium resolution structure of the complex [20], and computational studies [49]. It was proposed that if photochemical reactions are taking place in one RC within a dimer this trapping centre is closed in terms of accepting excitons from its LH1 complex. However, given connectivity between LH1 rings, there is ample time for sub-picosecond transfer of excitations to LH1 BChls in the other half of the dimer, providing a fresh opportunity for trapping by the other RC [20]. To examine whether there could be a requirement for such excitation sharing, we can refer to a quantitative treatment of bacterial photosynthesis, which considered all processes from absorption of sunlight to the production of ATP [49]. This analysis considered a range of incident light intensities, with one of these equivalent to 5% of full sunlight, i.e. 50 W/m^2^. Here, a chromatophore vesicle absorbs 1 860 photons per second, so each of the 24 monomer equivalent LH1 complexes in a chromatophore vesicle absorbs ∼80 photons per second, or one photon every 12.5 milliseconds [50]. Given that the turnover at the cytochrome *bc*_1_ complex is the limiting factor for cyclic electron flow [50,51], then under steady-state conditions the time constant for quinol production at the RC Q_B_ site must be limited by, and be equal to, the time constant for quinol oxidation by the cytochrome *bc*_1_ complex, which is 25 milliseconds [52]. Thus, there is a good chance that RC photochemistry will be engaged in the formation of quinols on millisecond timescales roughly equivalent to those for excitation of the LH1 antenna under low light conditions, i.e. 5% of full sunlight. It is therefore reasonable to envisage that, for higher rates of excitation of LH1 above 5% of full sunlight, there is an increasing chance of a mismatch between the input of light and the capacity of the cytochrome *bc*_1_ complex to process this energy. In these circumstances, excitons could migrate very rapidly from the half of the dimer with a ‘closed’ RC and be trapped at a potentially open RC in the other half. This proposition was examined using a series of *Rba. sphaeroides* strains containing monomeric or dimeric core complexes, also with low or high contents of the peripheral LH2 antenna complex [53]. A shorter LH1 excited state lifetime was observed in membranes containing dimeric core complexes compared with membranes with monomers, which was suggested to result from excitation sharing within a dimer. Thus, an exciton gains access to two RC traps, as also shown earlier by Comayras et al. [48]. The study in [53] measured a quantum efficiency of energy trapping of 93% in samples with dimeric RC-LH1 core complexes, and 90% with monomeric complexes. In summary, the structure of the RC-LH1 dimer shows, in molecular detail, the basis for connectivity between the LH1 rings, which could represent an adaptation that enhances the quantum efficiency of energy trapping in *Rba. sphaeroides*.

## Data Availability

The cryo-EM density map has been deposited in the World Wide Protein Data Bank (wwPDB) under accession code EMD-13590 and the coordinates have been deposited in the Protein Data Bank (PDB) under accession number 7PQD. The mass spectrometry proteomics data have been deposited to the ProteomeXchange Consortium via the PRIDE partner repository (http://proteomecentral.proteomexchange.org) with the data set identifier PXD028553.

## Open Access

Open access for this article was enabled by the participation of University of Sheffield in an all-inclusive *Read & Publish* pilot with Portland Press and the Biochemical Society under a transformative agreement with JISC.

## Abbreviations

AFM: atomic force microscopy
BChl: Bacteriochlorophyll
β-DDM: *n*-dodecyl β-D-maltoside
cryo-EM: cryogenic electron microscopy
*Rba.*: *Rhodobacter*
RC-LH1: reaction centre light-harvesting complex 1
SQDG: sulfoquinovosyl diacylglycerol

## References

[1] Qian, P., Croll, T.I., Swainsbury, D.J., Castro-Hartmann, P., Moriarty, N.W., Sader, K. et al. (2021) Cryo-EM structure of the *Rhodospirillum rubrum* RC-LH1 complex at 2.5 Å. Biochem. J. 478, 3253–3263 10.1042/BCJ20210511 34402504 PMC8454704

[2] Tani, K., Kanno, R., Ji, X.-C., Hall, M., Yu, L.-J., Kimura, Y. et al. (2021) Cryo-EM structure of the photosynthetic LH1-RC complex from *Rhodospirillum rubrum*. Biochemistry 60, 2483–2491 10.1021/acs.biochem.1c00360 34323477

[3] Yu, L.-J., Suga, M., Wang-Otomo, Z.-Y. and Shen, J.-R. (2018) Structure of photosynthetic LH1–RC supercomplex at 1.9 Å resolution. Nature 556, 209–213 10.1038/s41586-018-0002-9 29618814

[4] Bracun, L., Yamagata, A., Christianson, B.M., Terada, T., Canniffe, D.P., Shirouzu, M. et al. (2021) Cryo-EM structure of the photosynthetic RC-LH1-PufX supercomplex at 2.8-Å resolution. Sci. Adv. 7, eabf8864 10.1126/sciadv.abf8864 34134992 PMC8208714

[5] Swainsbury, D.J.K., Qian, P., Jackson, P.J., Faries, K.M., Niedzwiedzki, D.M., Martin, E.C. et al. (2021) Structures of *Rhodopseudomonas palustris* RC-LH1 complexes with open or closed quinone channels. Sci. Adv. 7, eabe2631 10.1126/sciadv.abe2631 33523887 PMC7806223

[6] Olsen, J.D., Martin, E.C. and Hunter, C.N. (2017) The PufX quinone channel enables the light-harvesting 1 antenna to bind more carotenoids for light collection and photoprotection. FEBS Lett. 591, 573–580 10.1002/1873-3468.12575 28130884 PMC5347945

[7] Farchaus, J.W., Gruenberg, H. and Oesterhelt, D. (1990) Complementation of a reaction center-deficient *Rhodobacter sphaeroides pufLMX* deletion strain in trans with *pufBALM* does not restore the photosynthesis-positive phenotype. J. Bacteriol. 172, 977–985 10.1128/jb.172.2.977-985.1990 2404961 PMC208526

[8] Barz, W.P., Francia, F., Venturoli, G., Melandri, B.A., Verméglio, A. and Oesterhelt, D. (1995) Role of PufX protein in photosynthetic growth of *Rhodobacter sphaeroides*. 1. PufX is required for efficient light-driven electron transfer and photophosphorylation under anaerobic conditions. Biochemistry 34, 15235–15247 10.1021/bi00046a032 7578139

[9] Barz, W.P., Verméglio, A., Francia, F., Venturoli, G., Melandri, B.A. and Oesterhelt, D. (1995) Role of the PufX protein in photosynthetic growth of *Rhodobacter sphaeroides*. 2. PufX is required for efficient ubiquinone/ubiquinol exchange between the reaction center Q_B_ site and the cytochrome *bc*_1_ complex. Biochemistry 34, 15248–15258 10.1021/bi00046a033 7578140

[10] Qian, P., Swainsbury, D.J.K., Croll, T.I., Salisbury, J.H., Martin, E.C., Jackson, P.J. et al. (2021) Cryo-EM structure of the *Rhodobacter sphaeroides* RC-LH1-PufXY monomer complex at 2.5 Å. Biochem. J., 478, 3775–3790 10.1042/BCJ20210631 34590677 PMC8589327

[11] Lavergne, J., Verméglio, A. and Joliot, P. (2009) Functional coupling between reaction centers and cytochrome bc1 complexes. In The Purple Phototrophic Bacteria (Hunter, C.N., Daldal, F., Thurnauer, M.C. and Beatty, J.T., eds), pp. 509–536, Springer Netherlands, Dordrecht

[12] Pugh, R.J., McGlynn, P., Jones, M.R. and Hunter, C.N. (1998) The LH1–RC core complex of *Rhodobacter sphaeroides*: interaction between components, time-dependent assembly, and topology of the PufX protein. Biochim. Biophys. Acta 1366, 301–316 10.1016/S0005-2728(98)00131-5 9814844

[13] Olsen, J.D., Adams, P.G., Jackson, P.J., Dickman, M.J., Qian, P. and Hunter, C.N. (2014) Aberrant assembly complexes of the reaction center light-harvesting 1 PufX (RC-LH1-PufX) core complex of *Rhodobacter sphaeroides* imaged by atomic force microscopy. J. Biol. Chem. 289, 29927–29936 10.1074/jbc.M114.596585 25193660 PMC4208002

[14] Francia, F., Wang, J., Venturoli, G., Melandri, B.A., Barz, W.P. and Oesterhelt, D. (1999) The reaction center−LH1 antenna complex of *Rhodobacter sphaeroides* contains one PufX molecule which is involved in dimerization of this complex. Biochemistry 38, 6834–6845 10.1021/bi982891h 10346905

[15] Francia, F., Wang, J., Zischka, H., Venturoli, G. and Oesterhelt, D. (2002) Role of the N- and C-terminal regions of the PufX protein in the structural organization of the photosynthetic core complex of *Rhodobacter sphaeroides*. Eur. J. Biochem. 269, 1877–1885 10.1046/j.1432-1033.2002.02834.x 11952789

[16] Ratcliffe, E.C., Tunnicliffe, R.B., Ng, I.W., Adams, P.G., Qian, P., Holden-Dye, K. et al. (2011) Experimental evidence that the membrane-spanning helix of PufX adopts a bent conformation that facilitates dimerisation of the *Rhodobacter sphaeroides* RC-LH1 complex through N-terminal interactions. Biochim. Biophys. Acta 1807, 95–107 10.1016/j.bbabio.2010.10.003 20937243

[17] Crouch, L.I., Holden-Dye, K. and Jones, M.R. (2010) Dimerisation of the *Rhodobacter sphaeroides* RC–LH1 photosynthetic complex is not facilitated by a GxxxG motif in the PufX polypeptide. Biochim. Biophys. Acta 1797, 1812–1819 10.1016/j.bbabio.2010.07.007 20646993

[18] Holden-Dye, K., Crouch, L.I. and Jones, M.R. (2008) Structure, function and interactions of the PufX protein. Biochim. Biophys. Acta 1777, 613–630 10.1016/j.bbabio.2008.04.015 18460337

[19] Qian, P., Bullough, P.A. and Hunter, C.N. (2008) Three-dimensional reconstruction of a membrane-bending complex: the RC-LH1-PufX core dimer of *Rhodobacter sphaeroides*. J. Biol. Chem. 283, 14002–14011 10.1074/jbc.M800625200 18326046

[20] Qian, P., Papiz, M.Z., Jackson, P.J., Brindley, A.A., Ng, I.W., Olsen, J.D. et al. (2013) Three-dimensional structure of the *Rhodobacter sphaeroides* RC-LH1-PufX complex: dimerization and quinone channels promoted by PufX. Biochemistry 52, 7575–7585 10.1021/bi4011946 24131108

[21] Siebert, C.A., Qian, P., Fotiadis, D., Engel, A., Hunter, C.N. and Bullough, P.A. (2004) Molecular architecture of photosynthetic membranes in *Rhodobacter sphaeroides*: the role of PufX. EMBO J. 23, 690–700 10.1038/sj.emboj.7600092 14765115 PMC381000

[22] Westerhuis, W.H., Sturgis, J.N., Ratcliffe, E.C., Hunter, C.N. and Niederman, R.A. (2002) Isolation, size estimates, and spectral heterogeneity of an oligomeric series of light-harvesting 1 complexes from *Rhodobacter sphaeroides*. Biochemistry 41, 8698–8707 10.1021/bi011663b 12093288

[23] Hsin, J., Gumbart, J., Trabuco, L.G., Villa, E., Qian, P., Hunter, C.N. et al. (2009) Protein-induced membrane curvature investigated through molecular dynamics flexible fitting. Biophys. J. 97, 321–329 10.1016/j.bpj.2009.04.031 19580770 PMC2711417

[24] Olsen, J.D., Tucker, J.D., Timney, J.A., Qian, P., Vassilev, C. and Hunter, C.N. (2008) The organization of LH2 complexes in membranes from *Rhodobacter sphaeroides*. J. Biol. Chem. 283, 30772–30779 10.1074/jbc.M804824200 18723509 PMC2662159

[25] Kumar, S., Cartron, M.L., Mullin, N., Qian, P., Leggett, G.J., Hunter, C.N. et al. (2017) Direct imaging of protein organization in an intact bacterial organelle using high-resolution atomic force microscopy. ACS Nano 11, 126–133 10.1021/acsnano.6b05647 28114766 PMC5269641

[26] Frese, R.N., Pamies, J.C., Olsen, J.D., Bahatyrova, S., van der Weij-de Wit, C.D., Aartsma, T.J. et al. (2008) Protein shape and crowding drive domain formation and curvature in biological membranes. Biophys. J. 94, 640–647 10.1529/biophysj.107.116913 17827217 PMC2157227

[27] Frese, R.N., Siebert, C.A., Niederman, R.A., Hunter, C.N., Otto, C. and van Grondelle, R. (2004) The long-range organization of a native photosynthetic membrane. Proc. Natl Acad. Sci. U.S.A. 101, 17994–17999 10.1073/pnas.0407295102 15601770 PMC539794

[28] Adams, P.G., Mothersole, D.J., Ng, I.W., Olsen, J.D. and Hunter, C.N. (2011) Monomeric RC-LH1 core complexes retard LH2 assembly and intracytoplasmic membrane formation in PufX-minus mutants of *Rhodobacter sphaeroides*. Biochim. Biophys. Acta 1807, 1044–1055 10.1016/j.bbabio.2011.05.019 21663730

[29] Sader, K., Matadeen, R., Castro Hartmann, P., Halsan, T. and Schlichten, C. (2020) Industrial cryo-EM facility setup and management. Acta Crystallogr. D Struct. Biol. 76, 313–325 10.1107/S2059798320002223 32254055 PMC7137108

[30] Zivanov, J., Nakane, T., Forsberg, B.O., Kimanius, D., Hagen, W.J.H., Lindahl, E. et al. (2018) New tools for automated high-resolution cryo-EM structure determination in RELION-3. eLife 7, e42166 10.7554/eLife.42166 30412051 PMC6250425

[31] Zheng, S.Q., Palovcak, E., Armache, J.-P., Verba, K.A., Cheng, Y. and Agard, D.A. (2017) Motioncor2: anisotropic correction of beam-induced motion for improved cryo-electron microscopy. Nat. Methods 14, 331–332 10.1038/nmeth.4193 28250466 PMC5494038

[32] Rohou, A. and Grigorieff, N. (2015) CTFFIND4: fast and accurate defocus estimation from electron micrographs. J. Struct. Biol. 192, 216–221 10.1016/j.jsb.2015.08.008 26278980 PMC6760662

[33] Grant, T., Rohou, A. and Grigorieff, N. (2018) cisTEM, user-friendly software for single-particle image processing. eLife 7, e35383 10.7554/eLife.35383 29513216 PMC5854467

[34] Pettersen, E.F., Goddard, T.D., Huang, C.C., Meng, E.C., Couch, G.S., Croll, T.I. et al. (2021) UCSF chimerax: Structure visualization for researchers, educators, and developers. Protein Sci. 30, 70–82 10.1002/pro.3943 32881101 PMC7737788

[35] Emsley, P. and Cowtan, K. (2004) Coot: model-building tools for molecular graphics. Acta Crystallogr. D Biol. Crystallogr. 60, 2126–2132 10.1107/S0907444904019158 15572765

[36] Croll, T. (2018) ISOLDE: a physically realistic environment for model building into low-resolution electron-density maps. Acta Crystallogr. D Struct. Biol. 74, 519–530 10.1107/S2059798318002425 29872003 PMC6096486

[37] Afonine, P.V., Poon, B.K., Read, R.J., Sobolev, O.V., Terwilliger, T.C., Urzhumtsev, A. et al. (2018) Real-space refinement in PHENIX for cryo-EM and crystallography. Acta Crystallogr. D Struct. Biol. 74, 531–544 10.1107/S2059798318006551 29872004 PMC6096492

[38] Flannery, S.E., Hepworth, C., Wood, W.H.J., Pastorelli, F., Hunter, C.N., Dickman, M.J. et al. (2021) Developmental acclimation of the thylakoid proteome to light intensity in Arabidopsis. Plant J. 105, 223–244 10.1111/tpj.15053 33118270 PMC7898487

[39] Chi, S.C., Mothersole, D.J., Dilbeck, P., Niedzwiedzki, D.M., Zhang, H., Qian, P. et al. (2015) Assembly of functional photosystem complexes in *Rhodobacter sphaeroides* incorporating carotenoids from the spirilloxanthin pathway. Biochim. Biophys. Acta 1847, 189–201 10.1016/j.bbabio.2014.10.004 25449968 PMC4331045

[40] Tucker, J.D., Siebert, C.A., Escalante, M., Adams, P.G., Olsen, J.D., Otto, C. et al. (2010) Membrane invagination in *Rhodobacter sphaeroides* is initiated at curved regions of the cytoplasmic membrane, then forms both budded and fully detached spherical vesicles. Mol. Microbiol. 76, 833–847 10.1111/j.1365-2958.2010.07153.x 20444085

[41] Tunnicliffe, R.B., Ratcliffe, E.C., Hunter, C.N. and Williamson, M.P. (2006) The solution structure of the PufX polypeptide from *Rhodobacter sphaeroides*. FEBS Lett. 580, 6967–6971 10.1016/j.febslet.2006.11.065 17161397

[42] Gorchein, A. (1973) Structure of the ornithine-containing lipid from *Rhodopseudomonas spheroides*. Biochim. Biophys. Acta 306, 137–141 10.1016/0005-2760(73)90218-X 4703568

[43] Zhang, X., Ferguson-Miller, S.M. and Reid, G.E. (2009) Characterization of ornithine and glutamine lipids extracted from cell membranes of *Rhodobacter sphaeroides*. J. Am. Soc. Mass Spectrometry 20, 198–212 10.1016/j.jasms.2008.08.017 PMC2779474 18835523

[44] Benning, C., Beatty, J.T., Prince, R.C. and Somerville, C.R. (1993) The sulfolipid sulfoquinovosyldiacylglycerol is not required for photosynthetic electron transport in Rhodobacter sphaeroides but enhances growth under phosphate limitation. Proc. Natl Acad. Sci. U.S.A. 90, 1561–1565 10.1073/pnas.90.4.1561 8434018 PMC45914

[45] Swainsbury, D.J.K., Scheidelaar, S., Foster, N., van Grondelle, R., Killian, J.A. and Jones, M.R. (2017) The effectiveness of styrene-maleic acid (SMA) copolymers for solubilisation of integral membrane proteins from SMA-accessible and SMA-resistant membranes. Biochim. Biophys. Acta 1859, 2133–2143 10.1016/j.bbamem.2017.07.011 PMC5593810 28751090

[46] Qian, P., Martin, E.C., Ng, I.W. and Hunter, C.N. (2017) The C-terminus of PufX plays a key role in dimerisation and assembly of the reaction center light-harvesting 1 complex from *Rhodobacter sphaeroides*. Biochim. Biophys. Acta 1858, 795–803 10.1016/j.bbabio.2017.06.001 PMC5538271 28587931

[47] Adams, P.G. and Hunter, C.N. (2012) Adaptation of intracytoplasmic membranes to altered light intensity in *Rhodobacter sphaeroides*. Biochim. Biophys. Acta 1817, 1616–1627 10.1016/j.bbabio.2012.05.013 22659614

[48] Comayras, F., Jungas, C. and Lavergne, J. (2005) Functional consequences of the organization of the photosynthetic apparatus in *Rhodobacter sphaeroides*. I. Quinone domains and excitation transfer in chromatophores and reaction center.antenna complexes. J. Biol. Chem. 280, 11203–11213 10.1074/jbc.M412088200 15632164

[49] Sener, M., Hsin, J., Trabuco, L.G., Villa, E., Qian, P., Hunter, C.N. et al. (2009) Structural model and excitonic properties of the dimeric RC-LH1-PufX complex from *Rhodobacter sphaeroides*. Chem. Phys. 357, 188–197 10.1016/j.chemphys.2009.01.003 20161332 PMC2678753

[50] Sener, M., Strumpfer, J., Singharoy, A., Hunter, C.N. and Schulten, K. (2016) Overall energy conversion efficiency of a photosynthetic vesicle. eLife 5, e09541 10.7554/eLife.09541 27564854 PMC5001839

[51] Cartron, M.L., Olsen, J.D., Sener, M., Jackson, P.J., Brindley, A.A., Qian, P. et al. (2014) Integration of energy and electron transfer processes in the photosynthetic membrane of *Rhodobacter sphaeroides*. Biochim. Biophys. Acta 1837, 1769–1780 10.1016/j.bbabio.2014.02.003 24530865 PMC4143486

[52] Crofts, A.R. (2004) The cytochrome *bc*_1_ complex: function in the context of structure. Annu. Rev. Physiol. 66, 689–733 10.1146/annurev.physiol.66.032102.150251 14977419

[53] Chenchiliyan, M., Timpmann, K., Jalviste, E., Adams, P.G., Hunter, C.N. and Freiberg, A. (2016) Dimerization of core complexes as an efficient strategy for energy trapping in *Rhodobacter sphaeroides*. Biochim. Biophys. Acta 1857, 634–642 10.1016/j.bbabio.2016.03.020 27013332

