# Supplementary material for: Cryo-EM structure of the dimeric Rhodobacter sphaeroides RC-LH1 core complex at 2.9 Å: the structural basis for dimerisation
Source: Biochem J. 2021 Nov 9;478(21):3923–37. doi: 10.1042/BCJ20210696 (PMC8652583; doi:10.1042/BCJ20210696)
Supplement: Supplementary Figures S1-S7 and Tables S1-S2 [file BCJ-478-3923-s1.pdf]

Supplementary material

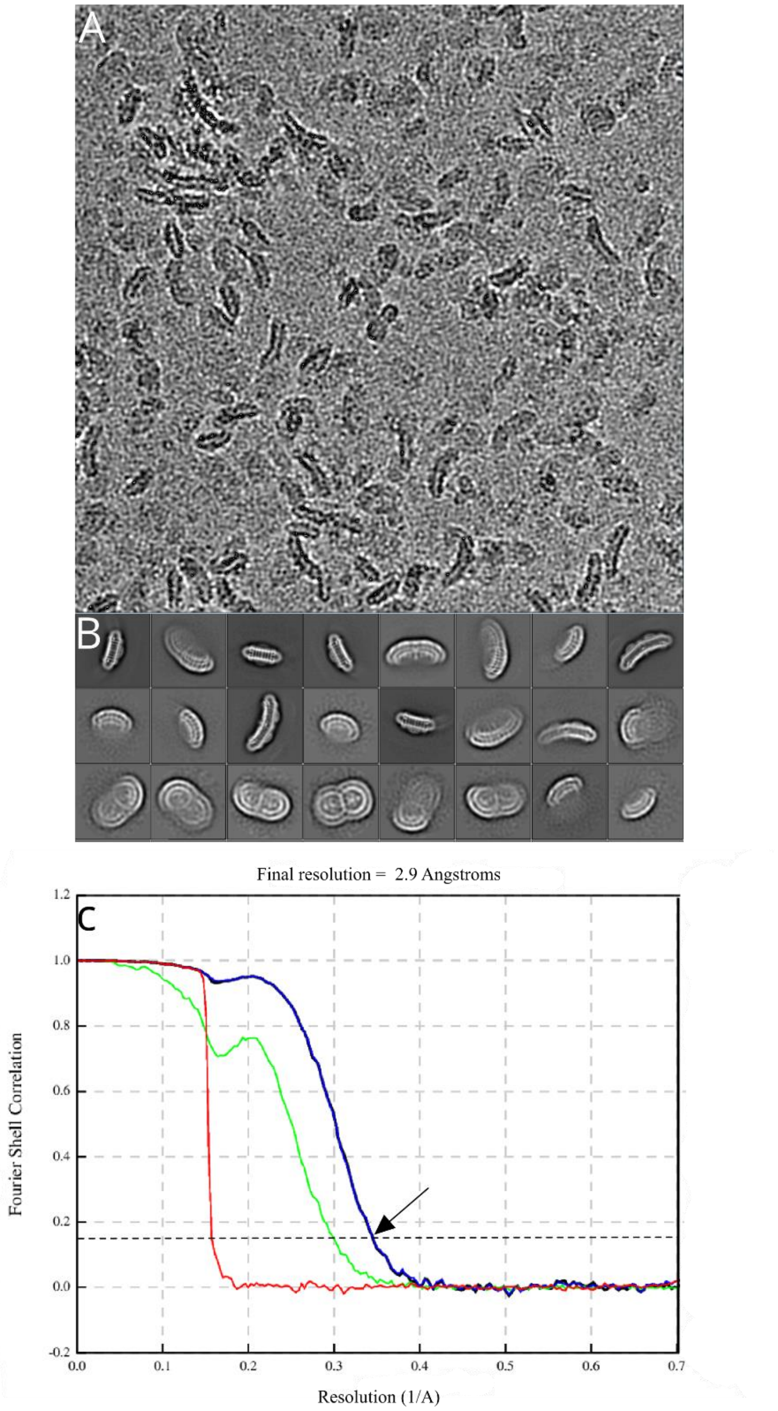

Figure S1. Cryo-EM image of RC-LH1-PufX dimer complexes from *Rba. sphaeroides* and resolution calculation of 3D map. (A) A motion corrected cryo-EM image of RC-LH1-PufX dimers embedded in vitrified water. The image was 20 Å low-pass filtered with a sigma contrast of 3.0 applied. The image size is 266 x 266 nm. (B) Selected 2D classes, showing different orientations of the RC-LH1-PufXYZ molecules in the vitrified ice layer. The box size is 30.5 nm. (C) Fourier shell correlation (FSC) curves of the CTF corrected (*black line*), masked (*blue solid line*), unmasked (*green line*), and phase randomized (*red line*) half-maps with C2 symmetry imposed. The dashed line shows 0.143 FSC, with an arrow pointing to the global resolution of the map at 2.9 Å.

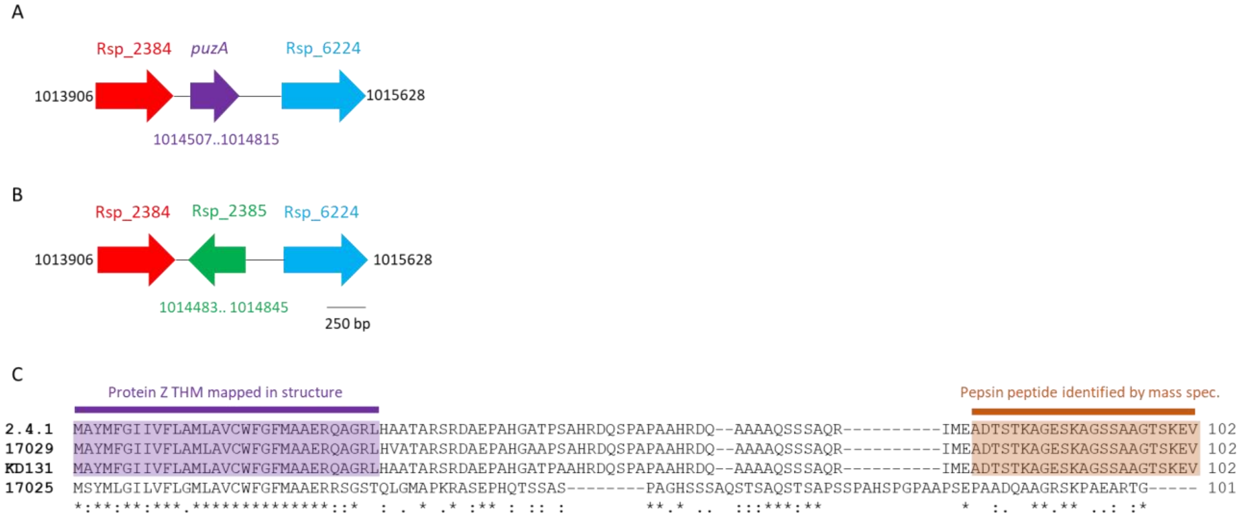

Figure S2. The protein-Z polypeptide. (A-B) The 1013906..1015628 region of the *Rba. sphaeroides* 2.4.1 genome showing the position of the putative '*puzA*' gene (A), which we predict is wrongly annotated as Rsp_2385 in the original genome sequence (B). Rsp_2384 encodes a putative stress protein and Rsp_6224 a putative membrane protein (phosphate-starvation-inducible E). (C) Multiple sequence alignment of the putative protein-Z homologs identified in the four *Rba. sphaeroides* (now also referred to as *Cereibacter sphaeroides*) strains in the KEGG database: *Rba. sphaeroides* 2.4.1, *Rba. sphaeroides* strain ATCC 17029, *Rba. sphaeroides* strain ATCC 17025 and *Rba. sphaeroides* strain KD131. The purple bar indicates the position of the conserved N-terminal region observed in our structure. The orange bar indicates the C-terminal pepsin peptide identified by mass spectrometry analysis of isolated RC-LH1 core complexes

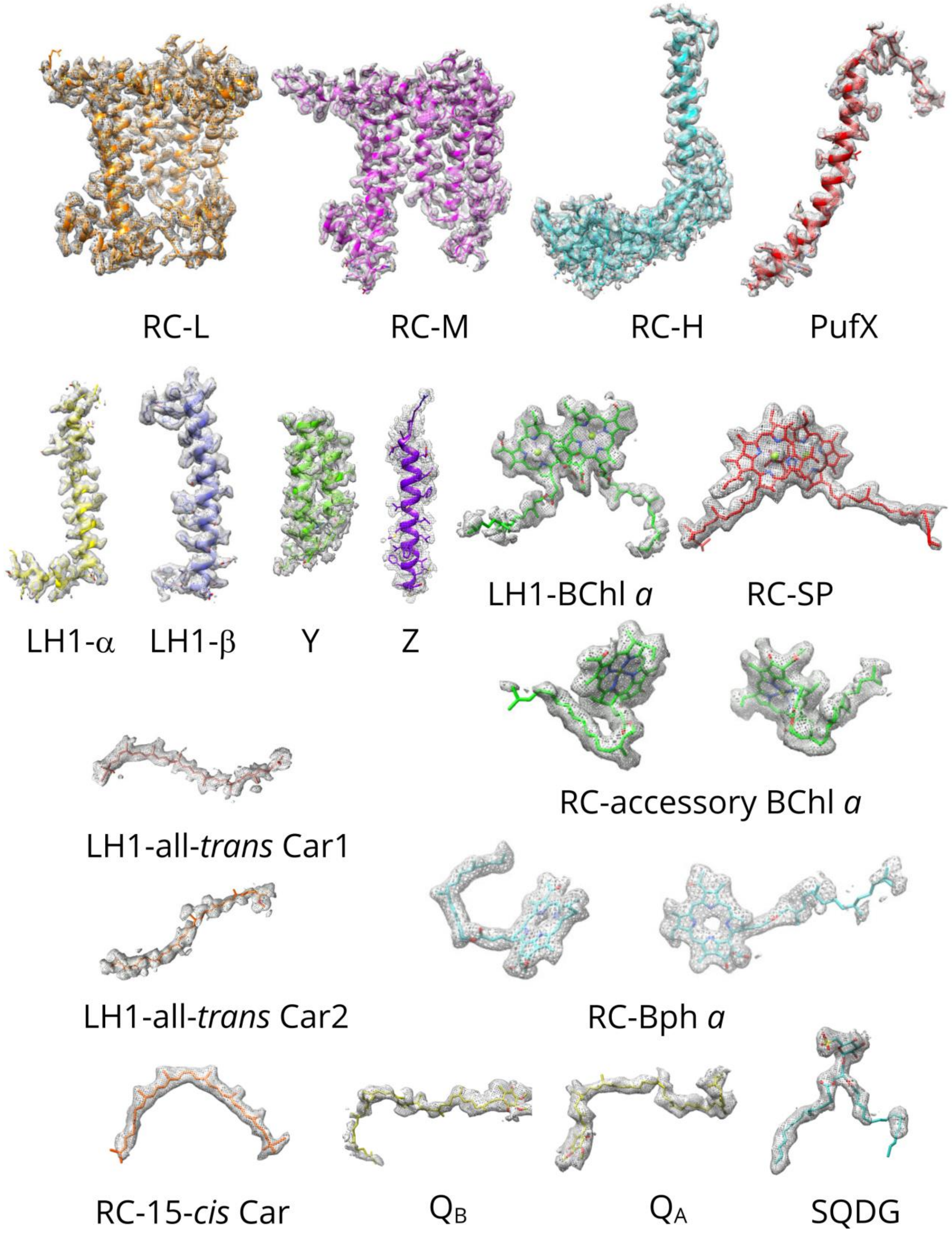

Figure S3. Cryo-EM densities and structural models of polypeptides and pigments in the dimeric *Rba. sphaeroides* RC-LH1-PufX complex. Atomic models of components of the complexes are fitted into their respective density maps, taken from the final refined model. RC-SP are the reaction centre special pair of BChls. Y and Z are protein-Y and protein-Z, respectively. RC-LH1-PufX has C2 symmetry; only the components in a monomer are shown. The colour code is the same as in Fig. 1.

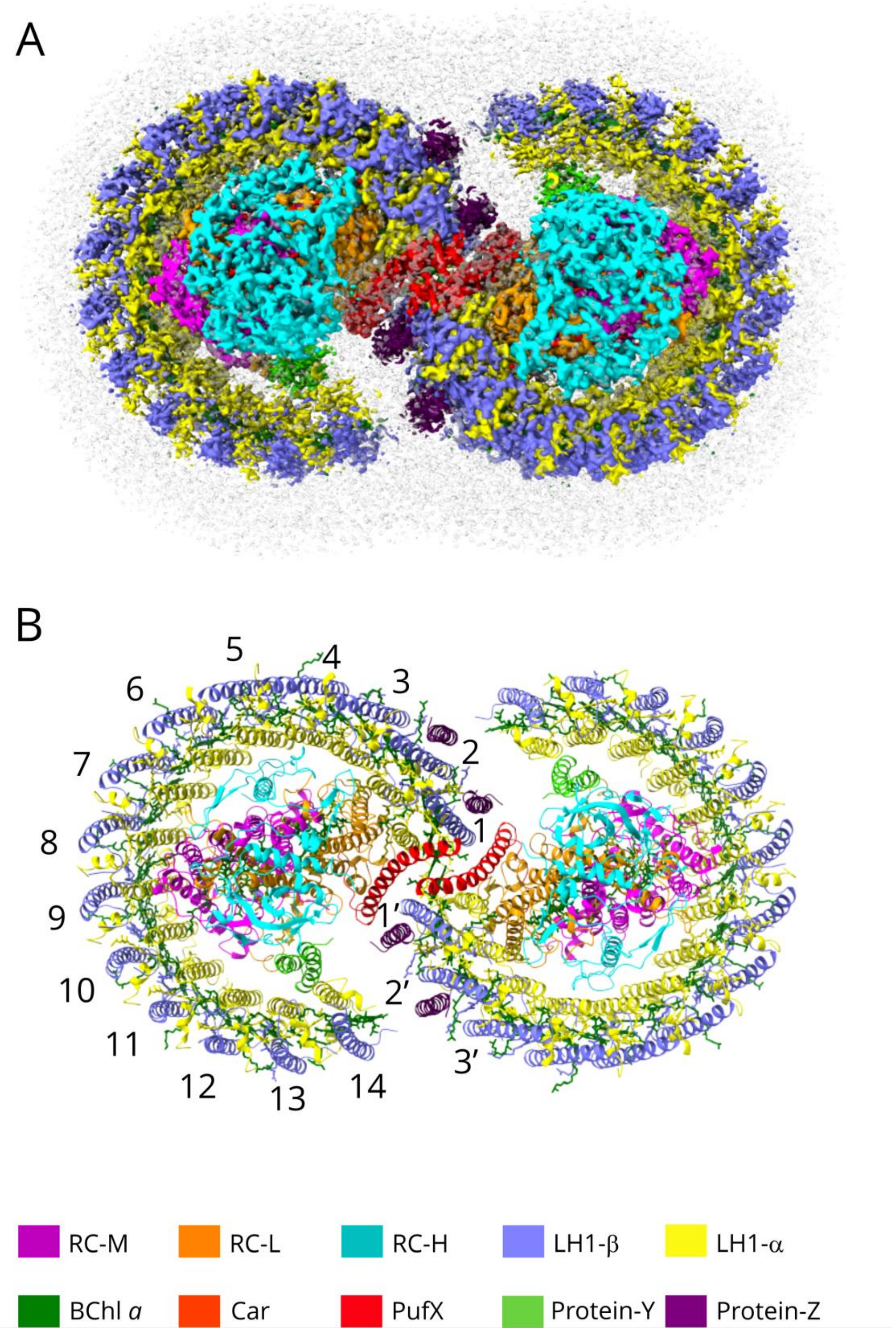

Figure S4. Cryo-EM structure of the dimeric RC-LH1 complex from *Rba. sphaeroides*. (A) View of the cytoplasmic face of the density map of the complex. (B) View as in (A), as a ribbon model; the LH1 subunits are numbered on the left hand side and subunits 1’-3’ on the other side. Subunits and cofactors are coloured as in the key at the bottom of the figure. Detergent and other disordered molecules are in grey.

RC-L

MALLSFERKYRVPGGTLVGGNLFDFWVGPFYVGFFGVATFFFAALGIILIAWSAVLQGTWNPQLISVYPPALE
YGLGGAPLAKGGLWQIITICATGAFVSWALREVEICRKLGIGYHIPFAFAFAILAYLTLVLFRPVMMGAWGYA
FPYGIWTHLDWVSNTGYTYGNFHYNPAHMIAISFFFTNALALALHGALVLSAANPEKGKEMRTPDHEDTFFRD
LVGYSIGTLGIHRLGLLLSLSAVFFSALCMIITGTIWFDQWVDWWQWWVKLPWWANIPGGING

RC-M

MAEYQNIFSQVQVRGPADLGMTEDVNLANRSGVGPFSTLLGWFGNAQLGPIYLGSLGVLSLFSGLMWFFTIGI
WFWYQAGWNPAVFLRDLFFFSLEPPAPEYGLSFAAPLKEGGLWLIASFFMFVAVWSWWGRTYLRAQALGMGKH
TAWAFLSAIWLWMVLGFIRPILMGSWSEAVPYGIFSHLDWTNNFSLVHGNLFYNPFHGLSIAFLYGSALLFAM
HGATILAVSRFGGERELEQIADRGTAAERAALFWRWTMGFNATMEGIHRWAIWMAVLVTLTGGIGILLSGTVV
DNWYVWGQNHGMAPLN

RC-H

MVGVTAFGNFDLASLAIYSFWIFLAGLIYYLQTENMREGYPLENEDGTPAANQGPFPLPKPKTFILPHGRGTL
TVPGPESEDRPIALARTAVSEGFPHAPTGDPMKDGVGPASWVARRDLPELDGHGHNKIKPMKAAAGFHVSAGK
NPIGLPVRGCDLEIAGKVVDIWVDIPEQMARFLEVELKDGSTRLLPMQMVKVQSNRVHVNALSSDLFAGIPTI
KSPTEVTLLEEDKICGYVAGGLMYAAPKRKSVVAAMLAEYA

LH1α

MSKFYKIWMIFDPRRVFVAQGVFLFLLAVMIHLILLSTPSYNWLEISAAKYNRVAVAE

LH1β

MADKSDLGYTGLTDEQAQELHSVYMSGLWLFSAVAIVAHLAVYIWRPWF

PufX

MADKTIFNDHLNTNPKTNLRLWVAFQMMKGAGWAGGVFFGTLLLIGFFRVVGRMLPIQENQAPAPNITGALET
GIELIKHLV

PufY

MPEVSEFAFRLMMAAVIFVGVGIMFAFAGGHWFVGLVVGGLVAAFFAATPNS

PufZ

MAYMFGIIVFLAMLAVCWFGFMAAERQAGRLHVATARSRDAEPAHGATPSSHRDQSPAPAAHRDQAAAAQSSS
AQRIMEADTSTKAGESKAGSSAAGTSKEV

Figure S5. Sequences of the proteins that comprise the dimeric RC-LH1-PufX complex. Residues in blue were modelled into the cryo-EM map. The numbering of RC-L, M residues in the main text follows the other entries for these RCs in the PDB, in omitting the first Met (shown here in black) and starting at the second residue, in each case Ala.

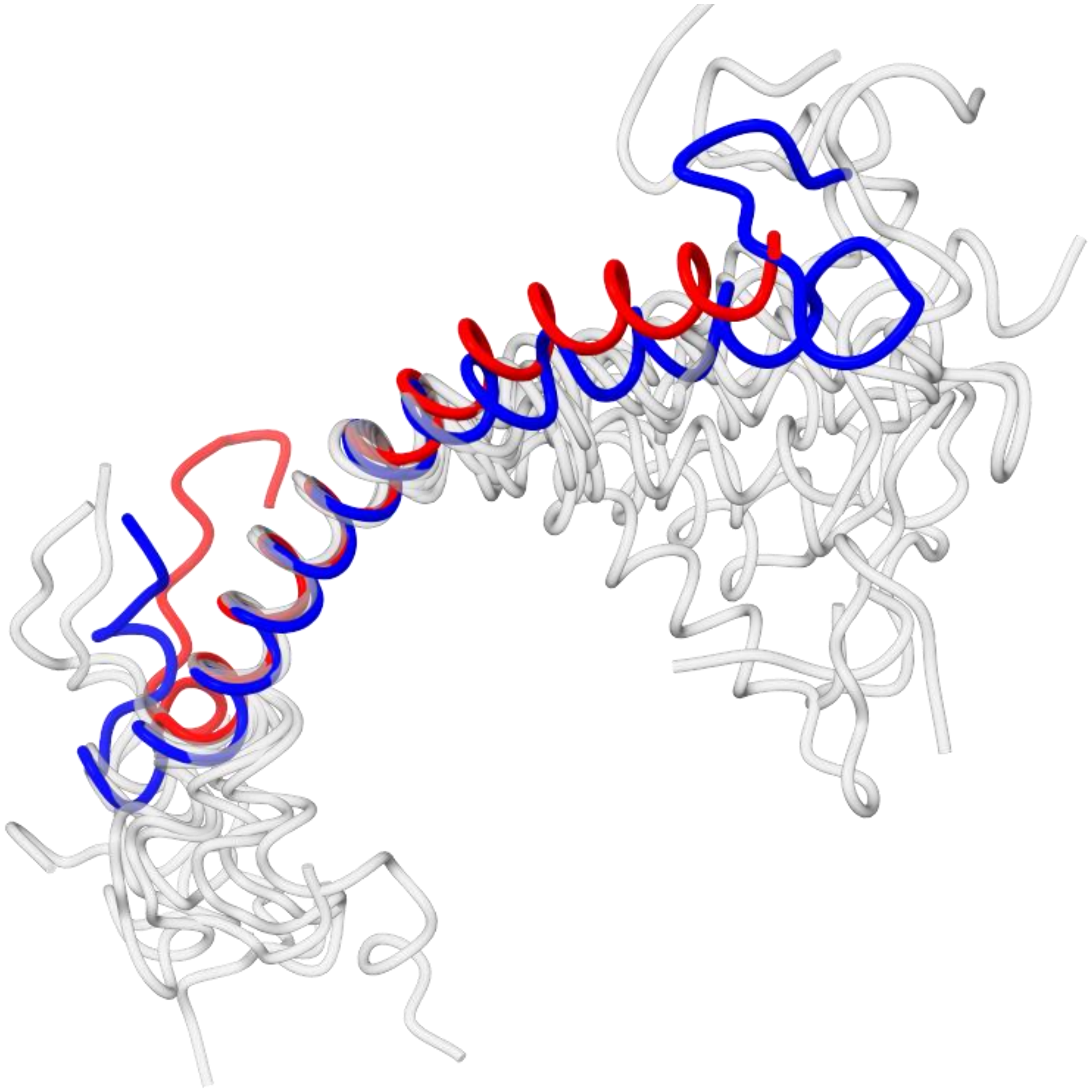

Figure S6. Overlaid NMR solution structures of PufX (grey) aligned to the full length of the transmembrane helix. The solution structure coloured in blue aligns to a limited degree with the PufX assembled within the dimer complex (red).

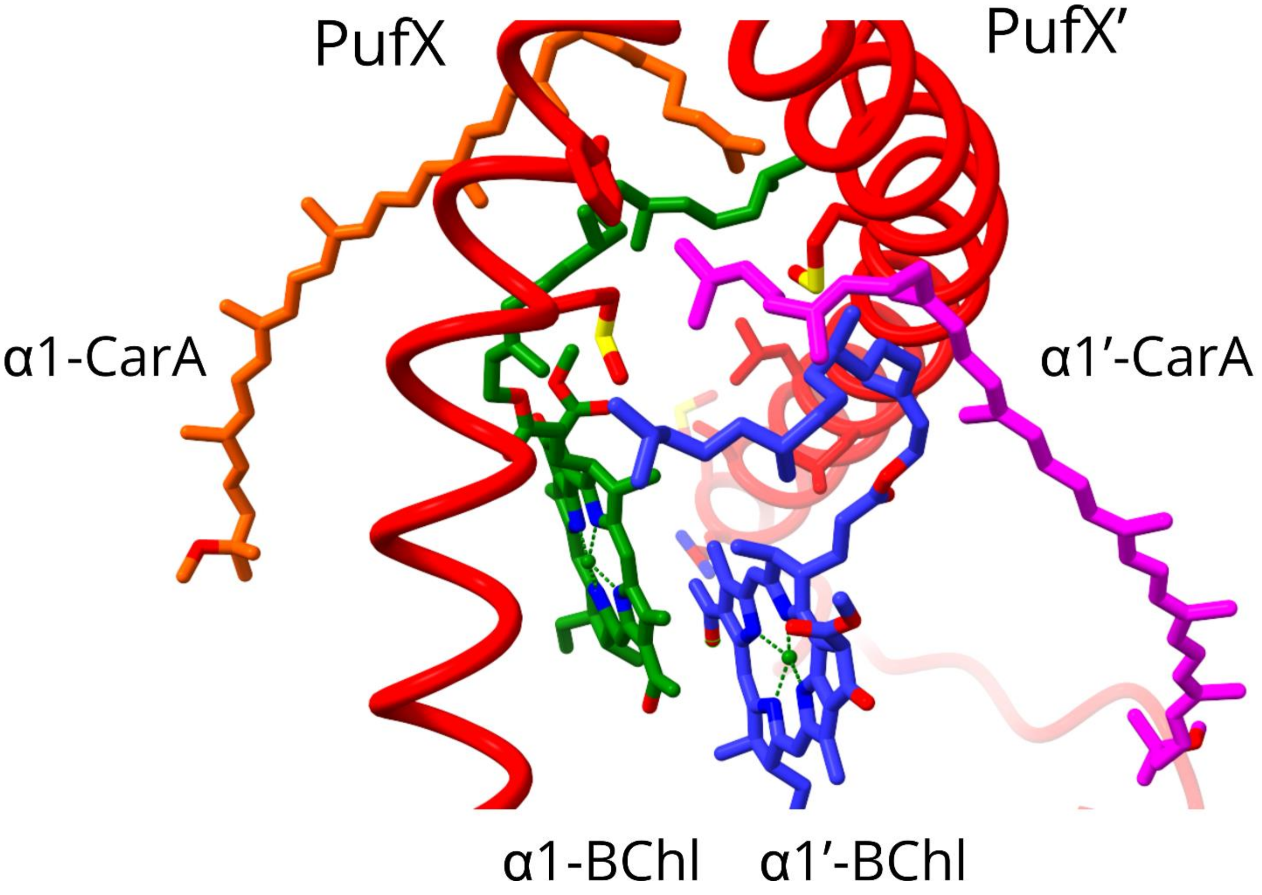

Fig. S7. The two central carotenoids and BChls at the dimer interface. The two PufX polypeptides incline towards each other, forming an arch that constrains and bows the CarA carotenoids, as well as the phytol tails of the two central BChls, α1-BChl and α1’-BChl.

Table S1. Cryo-EM data acquisition, model refinement and validation statistics.

Protein source	Photosynthetic bacterium
Data collection and processing	
Microscope	ThermoFisher Titan Krios G3i
Voltage (kV)	300
Camera	Falcon 4
Energy filter	No
Energy filter slit width	No
Magnification	120,000
Defocus range (µm)	-0.8 to -2.2
Mean defocus (µm)	-1.8
Pixel size (Å)	0.65
Electron flux (e^-/ Å^2/s)	3.71
Electron fluence (e^-/ Å^2)	45.36
Exposure time (sec/frame)	0.29
Electron fluence per frame (e/ Å^2/frame)	1.08
Number of frames per movie	42
Number of movies used	5058
Initial no. particle images	223786
Final no. particle images	58945
Symmetry imposed	C2
Local resolution range	2.8 to 3.1
Resolution of unmasked reconstruction (Å, FSC=0.143)	3.3
Resolution of masked reconstruction (Å, FSC=0.143)	2.9
Specimen temperature	~80K
Particle box size	(512 px)^2
Refinement and validation	
Refinement package	COOT, ISOLDE, PHENIX
Initial model	PDB 7PIL
Model resolution (Å, FSC=0.5)	2.9
Map sharpening B factor (Å^2)	-90.45
Model composition	
Non-hydrogen atoms	45992
Protein residues	4698
Molecular weight (kD)	646.1
Protein B factor (Å^2)	21.75
RMS deviations	
Bond length (Å)	0.0042
Bond angle (^{0})	0.82
Validation	
MolProbity score	0.97
Clashscore	1.63
Rotamer outliers (%)	0.36
EMRinger score	4.80
Cb deviations (%)	0.00
CaBLAM outliers (%)	1.0
Ramachandran plot	
Favoured (%)	97.75
Allowed (%)	1.89
Disallowed (%)	0.36
Ramachandran Z-score	-0.58
PDB ID	7PQD
EMDB ID	EMD-13590

Table S2. **A list of hydrogen bonds the RC-LH1 dimer complex, relating to Fig. 2B, Fig. 3D, E and Fig. 4B.**

Residue 1	Atom 1	Residue 2	Atom 2	Distance (Å)
		**LH1-Protein Z interactions (Fig. 2B)**		
Z1-Tyr3	OH (sc)	β1-Tyr43	O (bb)	3.0
Z1-Cys17	SG (sc)	β1-Ser32	OG (sc)	3.7
Z1-Glu25	OE2 (sc)	X-Arg20	NH2 (sc)	2.9
Z1-Ala28	O (bb)	X-Lys16	NZ (bb)	3.1
Z2-Ala2	O (bb)	β2-Tyr43	OH (sc)	3.0
Z2-Tyr3	OH (sc)	β2-Ile44	O (bb)	3.0
Z2-Cys17	SG (sc)	β2-Ser32	OG (sc)	3.4
Z2-Glu25	OE1 (sc)	α1-Ser2	OG (sc)	2.7
Z2-Ala28	O (bb)	α1-Lys3	NZ (sc)	2.8
		**Inter-monomer interactions (Fig. 3D,E)**		
α1-Leu44	O (bb)	β1′-Trp45	NE1 (sc)	3.3
X-Arg53	NH1 (sc)	β1′-Ile44	O (bb)	2.8
		**SQDG interactions (Fig. 4B)**		
L-Leu75	N (bb)	SQD100	O2	3.1
L-Leu75	N (bb)	SQD100	O3	3.3
L-Gly140	O (bb)	SQD100	O3	2.9
L-Gly140	O (bb)	SQD100	O4	3.6
X-Arg49	NH1 (sc)	SQD100	O7	2.8
X-Arg53	NH2 (sc)	SQD100	O7	3.0
X-Arg53	NE (sc)	SQD100	O8	3.3

The atom labels refer to those used in the accompanying structure file (PDB: 7PQD). bb – backbone; sc – sidechain.
